# Academic self-efficacy and online physical education learning engagement: the chain mediating roles of learning motivation and self-control

**DOI:** 10.3389/fpsyg.2026.1863252

**Published:** 2026-07-21

**Authors:** Wenzhi Yan, Guoyu Chen, Jinfu Wang

**Affiliations:** 1 School of Physical Education, Central China Normal University, Wuhan, China; 2 China Football College, Beijing Sport University, Beijing, China; 3 School of Physical Education, South China University of Technology, Guangzhou, China

**Keywords:** academic self-efficacy, chain mediation effect, learning motivation, online physical education learning engagement, self-control

## Abstract

**Background:**

Online physical education has become an important mode of instruction in higher education; however, insufficient student engagement remains a critical concern. This study aimed to examine the associations between academic self-efficacy and college students’ learning engagement in online physical education, and tested the serial mediating roles of learning motivation and self-control.

**Methods:**

A cluster sampling method was used to recruit 898 undergraduate students (502 males and 396 females) from two universities. Academic self-efficacy, learning motivation, self-control, and online physical education engagement were assessed using validated scales. Data were analyzed using descriptive statistics, correlation analysis, and tests of serial mediation.

**Results:**

The results indicated that: (1) academic self-efficacy was significantly and positively correlated with learning motivation, self-control, and online physical education learning engagement (*r* = 0.322, *p* < 0.001; *r* = 0.385, *p* < 0.001; *r* = 0.325, *p* < 0.001); (2) Learning motivation and self-control each played independent mediating roles in the relationship between academic self-efficacy and engagement in online physical education.; and (3) learning motivation and self-control jointly formed a serial mediation pathway between academic self-efficacy and online physical education engagement.

**Conclusion:**

These findings suggest that academic self-efficacy is associated with engagement in online physical education via pathways involving learning motivation and self-control. The results suggest that academic self-efficacy, learning motivation, and self-control are associated with higher levels of engagement in online physical education.

## Introduction

The rapid development of the digital economy has promoted the integration of information technology into education, positioning digital transformation as a central direction in higher education. In this context, online learning has evolved from a supplementary component of traditional classroom instruction into an independent mode of learning (Lin et al., 2021). The outbreak of COVID-19 further accelerated this transition (Sun et al., 2022). However, compared with the relatively smooth transition of theoretical courses, physical education faces more complex challenges in its shift to online formats. In online physical education, learning engagement is closely tied to students’ self-regulatory capabilities, including motivational maintenance and behavioral control (Puad et al., 2024). The absence of on-site supervision and interactive support means that the learning process is more dependent on individual self-regulation and more influenced by individual differences. As a key variable linking learning processes and outcomes, learning engagement provides a critical perspective for understanding the effectiveness of online physical education. Therefore, it is necessary to systematically examine the mechanisms underlying college students’ engagement in online physical education from a psychological perspective.

Learning engagement refers to a positive, persistent, and learning-related psychological state that individuals exhibit during the learning process, and it is typically characterized by three dimensions: vigor, dedication, and absorption (Membiela and González, 2023; Schaufeli et al., 2002). Previous studies have suggested that learning engagement not only reflects the quality of the learning process but also is considered an important factor associated with learning outcomes (Xu et al., 2020). In physical education, learning engagement demonstrates a more pronounced integration of physical and psychological dimensions, encompassing not only behavioral participation but also psychological components such as interest and emotional involvement (Yang et al., 2024). With the development of information technology, physical education courses have gradually expanded into online settings, accompanied by corresponding changes in the manifestation of learning engagement. In measuring online learning engagement, existing studies have increasingly incorporated behavioral data from learning platforms, such as login frequency and video viewing duration, in addition to self-report scales (Zhang et al., 2017). However, online learning engagement is essentially a multidimensional construct encompassing behavioral, cognitive, emotional, and social dimensions, with dynamic interactions among these dimensions (Singh et al., 2025), and therefore relying solely on objective behavioral indicators is insufficient to fully capture learners’ internal psychological engagement. An in-depth examination of the mechanisms underlying engagement in online physical education from a multidimensional perspective is of considerable theoretical and practical significance.

According to social cognitive theory, human behavior is shaped by the interaction between external environmental factors and internal cognitive processes, among which self-efficacy, as a core cognitive variable, plays a crucial role in the initiation and maintenance of behavior (Bandura, 1997). Self-efficacy refers to individuals’ beliefs in their capabilities to perform specific tasks (Elias and MacDonald, 2007), and its role has been widely demonstrated across domains such as academic performance, health behaviors, and behavioral regulation. In educational contexts, self-efficacy is often operationalized as academic self-efficacy, defined as learners’ subjective judgments of their ability to accomplish academic tasks (Xie et al., 2011). A substantial body of research indicates a robust positive association between academic self-efficacy and learning engagement. Individuals with high self-efficacy are more likely to sustain effort and exhibit greater concentration and persistence, whereas those with low self-efficacy are more prone to avoidance behaviors (Caraway et al., 2003). This association has also been observed in international online physical education settings. For example, Frikha et al. (2024) found that Saudi students’ competence perceptions—a construct closely related to self-efficacy—were significant predictors of academic achievement in online physical education. This relationship may be related to the motivational activation function of self-efficacy, which is associated with individuals’ sense of control over tasks, the use of effective learning strategies, and sustained effort in the face of difficulties (Bassi et al., 2007; Pajares, 1996). In online learning contexts, self-efficacy exhibits stronger context-dependent characteristics. Research suggests that online learning self-efficacy can be understood in terms of task competence, behavioral regulation, and environmental adaptation (Wright et al., 2012; Bandura, 1986), all of which may jointly relate to learners’ engagement in virtual environments. Empirical studies further show that learners with higher self-efficacy demonstrate greater engagement and completion rates in online courses (Galla et al., 2014; Pellas, 2014; Jung and Lee, 2018), whereas low self-efficacy may be negatively associated with engagement (Livingstone and Helsper, 2010). This positive association is particularly pronounced in online courses requiring a high degree of self-directed learning (Bahir and Wang, 2023), and online physical education represents a prototypical example. However, existing research has largely focused on general online learning contexts, with relatively limited attention to online physical education. Given the practical and context-dependent nature of physical education, academic self-efficacy may play a more critical role in this context. Accordingly, we propose: Hypothesis 1: Academic self-efficacy is positively associated with college students’ engagement in online physical education.

Learning motivation refers to the internal psychological force that is associated with the initiation, maintenance, and direction of goal-oriented behaviors in learning contexts, and is associated with the initiation and intensity of learning engagement (Chen and Liu, 2019). According to expectancy–value theory, individuals’ expectations of task success and their evaluations of task value are associated with the strength of their motivation (Wigfield and Eccles, 2000). Self-efficacy fundamentally reflects individuals’ confidence in their capabilities and their expectations of successful outcomes. Learners with higher self-efficacy tend to hold stronger beliefs in success, which in turn may support the development and maintenance of learning motivation (Cetin-Dindar, 2016). In online learning settings, individuals with higher self-efficacy typically report greater motivation and are more likely to invest effort even when facing challenging tasks (Park and Yun, 2018). As a key driver of behavioral activation, learning motivation is associated with engagement through its association with individuals’ effort allocation, attentional focus, and persistence (Duan and Hong, 2019). In online physical education, learning behaviors rely more heavily on intrinsic motivation, as online environments reduce external supervision and immediate feedback, making the learning process more dependent on self-regulation. Cross-sectional studies indicate that, in MOOC contexts, academic self-efficacy is significantly associated with learners’ engagement intentions and completion rates through pathways involving learning motivation. In physical education contexts, individuals with higher motivation typically report more frequent practice participation and longer durations of skill engagement (Hu et al., 2020; Huang and Cheng, 2024). By extension, the association between academic self-efficacy and online learning engagement may be linked to learning motivation, a pattern that has been shown to hold equally true in online physical education (Li, 2022). This provides direct contextual support for the present study. Based on the above, we propose: Hypothesis 2: Learning motivation mediates the relationship between academic self-efficacy and college students’ engagement in online physical education.

In addition to the motivational activation mechanism discussed above, another key factor associated with the persistence of learning behavior—self-control—has been identified as an important mediator between academic self-efficacy and learning engagement (Duckworth et al., 2019; Muraven and Baumeister, 2000), particularly in online physical education contexts characterized by limited external supervision and frequent distractions. According to the strength model of self-control, self-control is a depletable psychological resource, the level of which is associated with whether individuals can maintain goal-directed behavior in distracting environments (Muraven and Baumeister, 2000). Empirical evidence indicates that self-control is positively associated with learning engagement, which may involve inhibiting impulses, regulating attentional allocation, and sustaining goal-directed behavior (Wu et al., 2025). In online learning environments with limited external constraints, individuals with insufficient self-control resources are more susceptible to distractions such as entertainment and social activities, which may be associated with interruptions in learning behavior (Pintrich, 2004; Schunk and DiBenedetto, 2020).

On the other hand, academic self-efficacy is considered a key correlate of self-control. In academic contexts, social cognitive theory posits that self-efficacy is associated with individuals’ positive expectations of behavioral outcomes, which may be associated with greater willingness and capacity to mobilize self-control resources (Bandura, 1986; Bandura, 1977). Specifically, individuals with high academic self-efficacy are more likely to set clear learning goals and maintain behavioral consistency during task execution, thereby reporting higher levels of self-control (Zimmerman, 2000). Both cross-sectional and experimental studies demonstrate that, in online learning environments, academic self-efficacy is associated with less attentional distraction and behavioral procrastination, with its effects partially mediated by self-control (Meng, 2025; Roberts et al., 2025). Based on the above theoretical and empirical evidence, academic self-efficacy may be associated with college students’ engagement in online physical education through pathways involving self-control capacity. Accordingly, the following hypothesis is proposed: Hypothesis 3: Self-control mediates the relationship between academic self-efficacy and university students’ online physical education learning engagement.

From a dynamic perspective, learning behavior can be conceptualized as comprising two stages: initiation and sustained maintenance. Learning motivation is primarily associated with whether learning behavior is initiated, whereas self-control is associated with whether such behavior can be sustained. Functionally, these two constructs operate in a sequential manner: motivation may provide the impetus for behavioral initiation, whereas self-control may help maintain the stability of behavioral execution (Chen and Liu, 2019; Gollwitzer, 1990). Existing theories provide insights into the relationship between learning motivation and self-control. Self-determination theory posits that when learning motivation stems from intrinsic interest, autonomous choice, or value identification, individuals are more likely to mobilize and sustain self-control resources in pursuit of long-term goals. In contrast, controlled motivation is less effective in sustaining self-regulatory behavior (Ryan and Deci, 2000). Expectancy–value theory further suggests that individuals’ expectations of success and their evaluations of task value jointly are associated with the strength of their motivation. Higher levels of motivation may be associated with a greater likelihood of resisting immediate temptations and maintaining goal-directed behavior, which are core manifestations of self-control (Eccles and Wigfield, 2002). Eswaramoorthi et al. (2022) provided further support for this perspective, demonstrating that self-regulation-related factors significantly predicted course satisfaction and completion rates in a virtual physical education course designed with a learner engagement approach. Based on these perspectives, higher levels of learning motivation may be associated with greater willingness and capacity to employ self-control strategies in learning contexts. Empirical evidence further supports this relationship. Wang et al. found that the intrinsic value of academic activities is significantly associated with academic self-control and serves as a stronger and more reliable predictor than utility value (Wang et al., 2018). Pan et al. (2025) reported that intrinsic learning motivation is significantly associated with self-control among Chinese students. Xie et al. (2026) demonstrated that autonomous motivation is indirectly associated with academic performance through sequential pathways involving self-control and learning habits. Werner and Berkman (2024) argued that motivation plays a central role in the temporal dynamics of self-control, with intrinsically driven goal pursuit may be associated with when and how individuals exert self-control.

This relationship becomes more pronounced in online physical education contexts characterized by limited external constraints. Learners must not only initiate learning behaviors proactively but also sustain training routines in distracting environments. Therefore, learning motivation not only is associated with behavioral initiation but may also be associated with sustained engagement through pathways involving self-control. Based on the above, learning motivation may be considered a key correlate of self-control and may function as part of a sequential pathway between academic self-efficacy and engagement in online physical education. Accordingly, we propose: Hypothesis 4: Learning motivation and self-control jointly mediate the relationship between academic self-efficacy and college students’ engagement in online physical education in a sequential manner.

**Figure 1:**
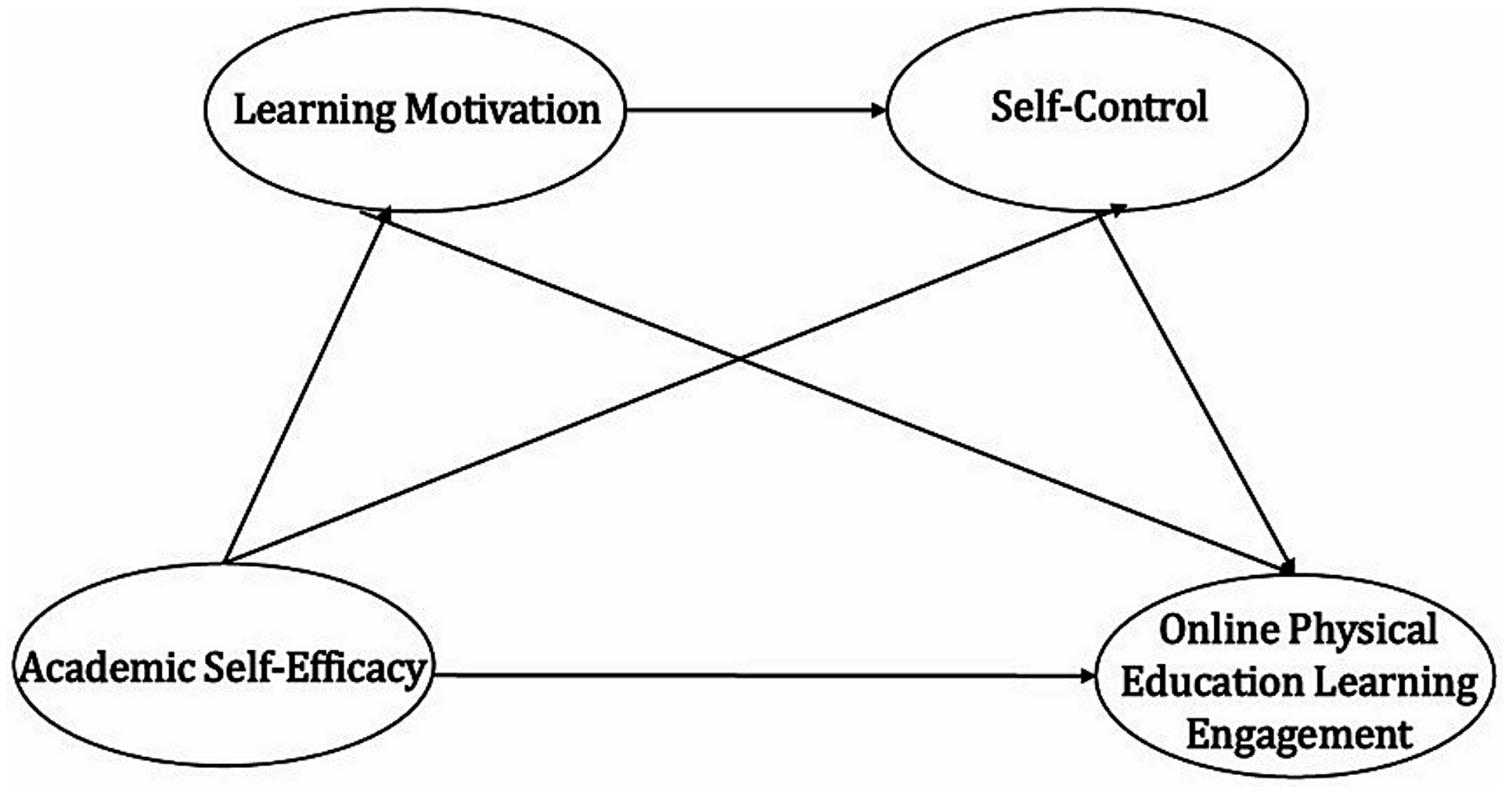
The hypothesized sequential mediation model of the present study.

In summary, existing research indicates that academic self-efficacy is significantly associated with college students’ learning behaviors and may also be positively associated with physical education behaviors. However, studies that systematically examine these mechanisms from the perspective of physical education engagement remain limited, particularly those incorporating both learning motivation and self-control within a unified framework. It remains unclear whether academic self-efficacy is indirectly associated with engagement in online physical education through pathways involving learning motivation and self-control, and whether these two variables function as sequential mediators. These issues warrant further investigation. Accordingly, this study draws on social cognitive theory, expectancy–value theory, and the strength model of self-control to construct a sequential mediation model incorporating learning motivation and self-control (see Figure 1). The study aims to examine the mechanisms underlying engagement formation in online physical education among college students and to contribute to the empirical understanding of the relationship between academic self-efficacy and physical education behaviors.

## Materials and methods

### Participants and procedure

Prior to data collection, *a priori* sample size estimation was conducted to ensure the stability of structural equation modeling (SEM) estimates. According to established guidelines, the sample size should be at least 10 times the number of observed variables (Lu et al., 2026). The present study included 44 observed variables, corresponding to a minimum sample size of 440. Given the higher sample size requirements for complex models (e.g., multiple or serial mediation), prior research recommends a sample size exceeding 500 (Kline, 2016). Accordingly, the target sample size was set at over 600, with an additional 20% buffer to account for invalid responses, resulting in an expected total of at least 720 collected questionnaires.

Data were collected using a cluster sampling method. Two key national universities directly affiliated with the Ministry of Education of China were selected: South China University of Technology (Guangzhou, Guangdong Province, South China) and Central China Normal University (Wuhan, Hubei Province, Central China). These two institutions differ in their disciplinary composition and institutional orientation. South China University of Technology has particular strengths in engineering, science, and management disciplines, while Central China Normal University has particular strengths in education, humanities, and social sciences. This selection was intended to enhance the diversity of the sample by covering both southern and central regions of China and incorporating different disciplinary traditions, thereby improving the external validity of the findings. Non-physical education major students from various disciplinary backgrounds were recruited at the class level. Specifically, the research team first contacted course instructors of relevant classes across different colleges. After obtaining instructors’ support, research assistants explained the purpose of the survey, the voluntary nature of participation, and confidentiality measures to students during class sessions, after which the questionnaires were distributed via an online platform and administered with the assistance of academic advisors. The inclusion criteria were: (1) currently enrolled full-time undergraduates at the two participating universities; (2) non-physical education majors; (3) ownership of a smartphone with access to the online survey platform; (4) absence of any congenital or chronic physical conditions that would impede regular physical activity; (5) sufficient proficiency in Mandarin Chinese to comprehend the questionnaire items and provide valid responses; (6) had participated in online physical education courses during the previous semester. Participants who failed to meet any of the above criteria were excluded from the final sample. This procedure yielded 1,025 initial responses. To ensure data quality, the dataset was screened as follows: responses with more than 10% missing data were excluded (*n* = 47); questionnaires showing evident response patterns (e.g., long strings of identical answers) were removed (*n* = 32); and responses failing reverse-coded item consistency checks were excluded (*n* = 48). After data cleaning, 898 valid responses were retained, yielding an effective response rate of 87.61%. The mean age of participants was 20.37 years (SD = 1.67). Sample characteristics are presented in Table 1. This study adhered to the ethical principles of the Declaration of Helsinki and was approved by the Ethics Committee of Central China Normal University (approval No. CCNU-IRB-202512014b). All participants provided informed consent electronically prior to participation.

**Table 1:** Demographic characteristics of the participants.

Variables	Category	*N*	Percentage (%)
Gender	Male	502	55.9
	Female	396	44.1
Grade	Freshman	214	23.8
	Sophomore	225	25.1
	Junior	230	25.6
	Senior	229	25.5
Place of origin	Rural	446	49.7
	Urban	452	50.3
Field of study	Humanities and social sciences	465	51.8
	Science and engineering	433	48.2

### Measures

#### Instrument adaptation process

All scales used in this study were originally developed for general academic or classroom contexts. To adapt them to online physical education, we followed a consistent procedure: (1) contextual wording adjustments (e.g., replacing general references with “online physical education” or “online physical education courses”); (2) review by the research team to confirm clarity and appropriateness; and (3) psychometric evaluation including confirmatory factor analysis and reliability testing. No items were deleted, added, or substantially reworded. Detailed adaptation procedures and psychometric properties for each scale are reported in the respective subsections below.

#### Online physical education learning engagement

Learning engagement refers to a positive psychological state exhibited during the learning process, encompassing three core components: cognitive engagement, emotional engagement, and behavioral engagement. This study adapted the Remote Learning Engagement Scale developed by Sun et al. to the specific context of online physical education (Sun and Rueda, 2012). The adaptation was limited to contextual wording adjustments; the core item content and structure were retained without modification. The revised scale comprises three dimensions—cognitive, emotional, and behavioral engagement—with a total of 14 items, rated on a 5-point Likert scale (1 = strongly disagree to 5 = strongly agree), where higher scores indicate greater engagement. During administration, participants were provided with a contextualized instruction: “In the context of online physical education, individuals may demonstrate varying levels of learning engagement. Please select the option that best reflects your experience.” Confirmatory factor analysis (CFA) indicated a good model fit (*χ*^2^ = 341.438, *χ*^2^/df = 3.925, TLI = 0.970, CFI = 0.975, SRMR = 0.033, RMSEA = 0.057). For the three dimensions of the online physical education learning engagement scale, the composite reliability (CR) values were 0.893, 0.957, and 0.939, and the average variance extracted (AVE) values were 0.736, 0.762, and 0.768, respectively, all exceeding the acceptable thresholds and indicating good convergent validity (Fornell and Larcker, 1981). Discriminant validity was assessed using the Fornell–Larcker criterion. The square roots of the AVE for the three dimensions ranged from 0.858 to 0.876, all exceeding the inter-dimension correlations (which ranged from 0.700 to 0.709), indicating good discriminant validity among the dimensions. Reliability analysis demonstrated high internal consistency (Cronbach’s *α* = 0.959; Guttman split-half reliability = 0.905).

#### Academic self-efficacy

Academic self-efficacy refers to individuals’ beliefs in their capability to organize and execute actions required to achieve intended outcomes in specific learning tasks. This study assessed academic self-efficacy using the subscale from the Adaptive Learning Patterns Scale revised by Chen et al., which comprises five items (Chen et al., 2016). To align with the study context, the items were contextually adapted by incorporating references to “online course learning” (e.g., “I believe I can master the knowledge and skills learned in online physical education classes”). The adaptation was limited to contextual wording adjustments; the core item content and structure were retained without modification. All items were rated on a 5-point Likert scale, with higher scores indicating greater academic self-efficacy. CR was 0.943, and the AVE was 0.768, indicating satisfactory convergent validity and internal consistency reliability (Fornell and Larcker, 1981). Confirmatory factor analysis further supported an acceptable fit of the single-factor model (*χ*^2^ = 17.586, *χ*^2^/df = 5.862, TLI = 0.985, CFI = 0.988, SRMR = 0.026, RMSEA = 0.074). Cronbach’s *α* for this scale was 0.942.

#### Learning motivation

Learning motivation refers to the intrinsic drive that activates and sustains behavior toward specific learning goals. This study assessed learning motivation using the scale revised by Long and Liu, which comprises five items (Long and Liu, 2016). To align with the context of online physical education, the items were contextually adapted (e.g., “In online physical education courses, I do not want to fall behind other students”). The adaptation was limited to contextual wording adjustments; the core item content and structure were retained without modification. All items were rated on a 5-point Likert scale (1 = strongly disagree to 5 = strongly agree), with higher scores indicating greater learning motivation. CFA indicated a good fit of the single-factor model (*χ*^2^ = 10.221, *χ*^2^/df = 2.044, TLI = 0.926, CFI = 0.945, SRMR = 0.011, RMSEA = 0.034). In the present sample, CR for this scale was 0.890, and the AVE was 0.620, indicating good convergent validity (Fornell and Larcker, 1981). Reliability analysis demonstrated good internal consistency (Cronbach’s *α* = 0.891; Guttman split-half reliability = 0.844), indicating that the scale reliably measures students’ online learning motivation.

#### Self-control

Self-control refers to the capacity to regulate immediate impulses and align behavior with long-term goals. This study assessed self-control using the Self-Control Scale originally developed by Tangney and revised by Tan, which comprises 19 items across five dimensions: impulse control, healthy habits, resistance to temptation, focus on work, and moderation in entertainment (Tan and Guo, 2008). Items 1, 5, 11, and 14 were positively keyed, while the remaining items were reverse-coded. All items were rated on a 5-point Likert scale (1 = strongly disagree to 5 = strongly agree), with higher scores indicating greater self-control. CFA indicated a good fit of the five-factor model (*χ*^2^ = 856.602, *χ*^2^/df = 6.299, TLI = 0.927, CFI = 0.942, SRMR = 0.047, RMSEA = 0.077), supporting the structural validity of the scale. The five dimensions had CR values of 0.913, 0.755, 0.813, 0.755, and 0.842, respectively, and AVE values of 0.636, 0.503, 0.521, 0.494, and 0.641, respectively. Although the AVE for the focus on work dimension was slightly below 0.50, its CR value was 0.755, exceeding the acceptable threshold of 0.70, and the AVE values for the remaining four dimensions were all above 0.50. According to Fornell and Larcker’s recommendation (Fornell and Larcker, 1981), when CR reaches an acceptable level, an AVE slightly below 0.50 is still acceptable. Therefore, the self-control scale demonstrated acceptable convergent validity. Discriminant validity was assessed using the Fornell–Larcker criterion. The square roots of the AVE for each dimension ranged from 0.703 to 0.801, all exceeding the inter-dimension correlations (ranged from 0.41 to 0.56), indicating adequate discriminant validity among the five dimensions. Reliability analysis demonstrated high internal consistency (Cronbach’s *α* = 0.957; Guttman split-half reliability = 0.950). All subscale Cronbach’s α values exceeded 0.75, indicating that the scale reliably measures participants’ self-control.

#### Demographic variables

In addition to the primary research variables, the study also collected demographic information including age, gender, grade level, academic discipline, and place of origin, which were subsequently treated as control variables in the analyses.

### Statistical analysis

All statistical analyses were conducted using SPSS 27.0 and AMOS 26.0. Descriptive statistics were first computed to summarize sample characteristics and the distributions of the main study variables. Common method bias was assessed using Harman’s single-factor test, and normality was examined based on skewness and kurtosis values. Pearson correlation analysis was then performed to examine the relationships among the variables. SEM was subsequently employed to test the hypothesized relationships. A sequential mediation model was specified, with academic self-efficacy as the exogenous variable, online physical education learning engagement as the endogenous variable, and learning motivation and self-control as sequential mediators. Demographic variables were included as covariates in all models. The mediating effects were tested using a bias-corrected bootstrap procedure with 5,000 resamples. Statistical significance was determined based on 95% confidence intervals. Model fit was evaluated using multiple indices, including the chi-square to degrees of freedom ratio (*χ*^2^/df), the comparative fit index (CFI), the Tucker–Lewis index (TLI), the standardized root mean square residual (SRMR), and the root mean square error of approximation (RMSEA). Values of CFI and TLI ≥ 0.90 and RMSEA and SRMR ≤ 0.08 were considered indicative of acceptable model fit (Wang et al., 2025). All statistical tests were two-tailed, with *p* < 0.05 indicating statistical significance.

## Results

### Common method bias analysis

Given that all data were collected using self-report measures, Harman’s single-factor test was conducted to assess potential common method bias. The unrotated exploratory factor analysis revealed multiple factors with eigenvalues greater than 1, with the first factor accounting for 37.41% of the total variance. This value is below the recommended threshold of 40% (Wang et al., 2024). However, Harman’s single factor test is considered a comparatively weak approach for assessing common method variance (Podsakoff et al., 2003). To address this limitation, we further employed the unmeasured latent variable technique as a supplementary test. Comparing models with and without a latent method factor revealed negligible improvement in fit indices (ΔCFI < 0.01, ΔTLI < 0.01, ΔRMSEA < 0.005, ΔSRMR < 0.005) (Tang and Wen, 2020). This result indicates that common method bias does not pose a major threat to the findings.

### Participant characteristics

A total of 898 participants were included in this study (see Table 1). Regarding gender distribution, 502 participants (55.9%) were male and 396 (44.1%) were female, indicating a slightly higher proportion of males. In terms of academic year, the sample was relatively evenly distributed, with 214 freshman students (23.8%), 225 sophomore students (25.1%), 230 junior students (25.6%), and 229 senior students (25.5%), suggesting no substantial imbalance across grade levels. With respect to place of origin, 446 participants (49.7%) were from rural areas and 452 (50.3%) were from urban areas, indicating a balanced distribution. In terms of academic discipline, 465 participants (51.8%) were from humanities and social sciences, while 433 (48.2%) were from science and engineering, reflecting a relatively even distribution across fields of study.

### Descriptive statistics and correlation analysis

Descriptive statistics and correlation analyses were conducted using SPSS 27.0. The means, standard deviations, and correlation coefficients of all variables are presented in Table 2. The results indicated that academic self-efficacy (*M* = 13.83, *SD* = 6.11), learning motivation (*M* = 14.89, *SD* = 5.75), self-control (*M* = 55.30, *SD* = 19.58), and online physical education learning engagement (*M* = 42.34, *SD* = 16.84) all exhibited variability across individuals. Correlation analysis revealed that all major variables were significantly and positively correlated (*r* = 0.322–0.444, all *p* < 0.001). Specifically, academic self-efficacy was significantly correlated with learning motivation (*r* = 0.322), self-control (*r* = 0.385), and online physical education learning engagement (*r* = 0.325). In addition, learning motivation was significantly correlated with self-control (*r* = 0.406) and online physical education learning engagement (*r* = 0.353). Overall, these findings provide preliminary support for the hypothesized relationships and justify further model testing.

**Table 2:** Descriptive statistics and correlation analysis (*N* = 898).

Variables	M	SD	1	2	3	4
1. Academic self-efficacy	13.83	6.108	1			
2. Learning motivation	14.89	5.752	0.322***	1		
3. Self-control	55.30	19.58	0.385***	0.406***	1	
4. Online physical education learning engagement	42.34	16.84	0.325***	0.353***	0.444***	1

M, mean score; SD, standard deviation. ^***^p < 0.001.

### Testing for chain mediation effects

#### Model fit

After establishing the reliability and validity of the measurement instruments, the structural model was evaluated while controlling for relevant covariates (see Table 3). The results indicated that all model fit indices fell within acceptable ranges. Specifically, the chi-square to degrees of freedom ratio was within an acceptable range (*χ*^2^/df < 5), the CFI and TLI both exceeded 0.90, and the residual-based indices, including the RMSEA and the SRMR, were below 0.08. These findings suggest that the proposed model provides an adequate fit to the data, supporting subsequent analyses of the structural relationships.

**Table 3:** Goodness-of-fit indices for the structural model.

Category	Fit index	Fit indices	Recommended threshold
Absolute fit indices	SRMR	0.016	<0.08
	RMSEA	0.054	<0.08
Incremental fit indices	TLI	0.979	>0.90
	CFI	0.986	>0.90
Parsimonious fit index	*χ^2^*/df	3.605	<5

CFI, comparative fit index; TLI, Tucker–Lewis index; RMSEA, root mean square error of approximation; SRMR, standardized root mean square residual.

### Direct effects

**Figure 2:**
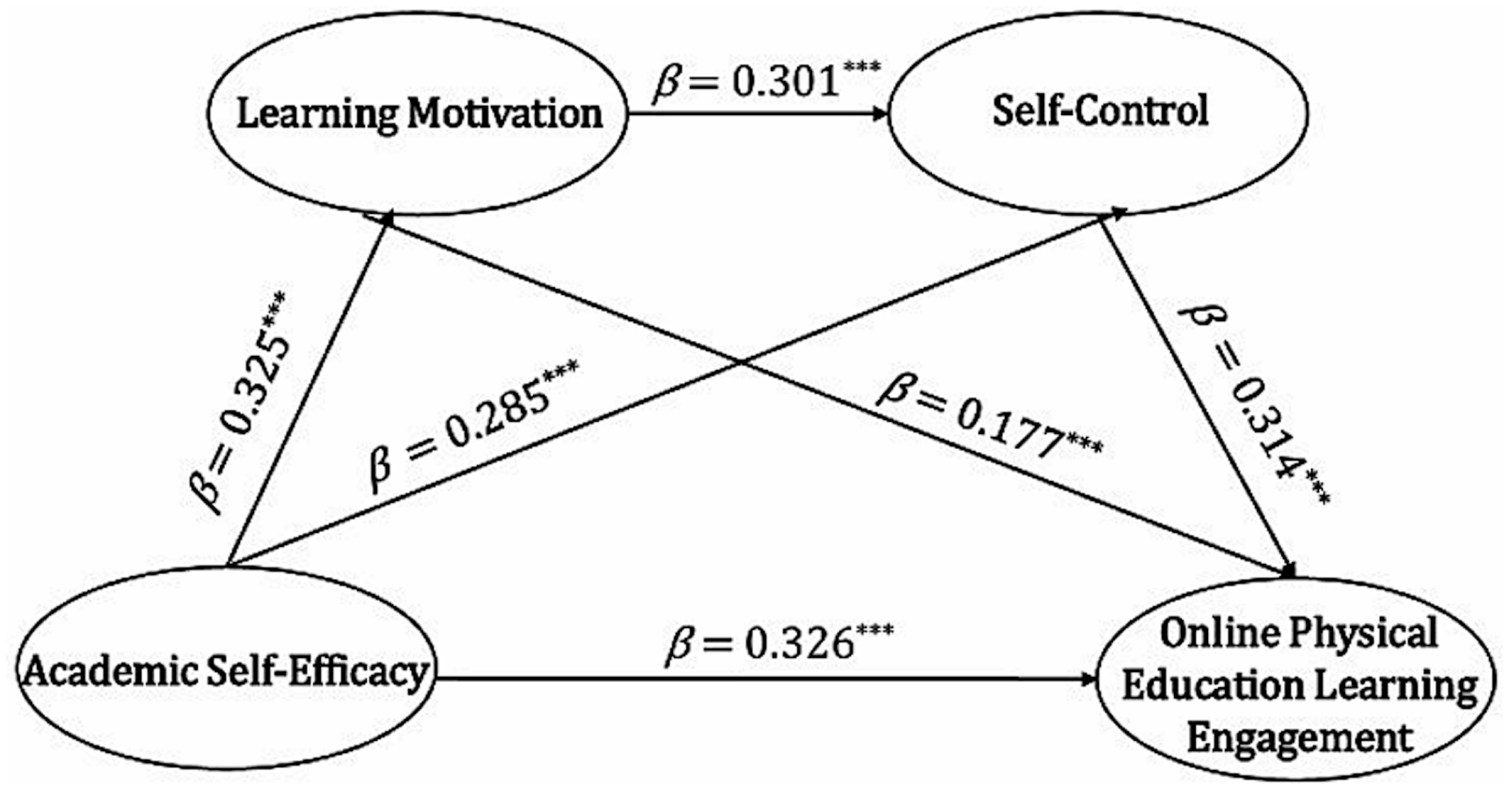
Final model of the present study. *β*, standardized coefficient; ***p < 0.001.

Given the satisfactory model fit, structural path estimates were obtained using maximum likelihood estimation in AMOS (see Table 4). Standardized path coefficients were used to indicate the direction and strength of the relationships among variables, and the model was visually presented in Figure 2. The results showed that, after controlling for relevant covariates, academic self-efficacy had a significant positive direct effect on online physical education learning engagement (*β* = 0.326, *p* < 0.001), indicating that higher levels of academic self-efficacy were associated with greater engagement. In addition, all paths involved in the mediating processes were statistically significant. Specifically, in the first stage of mediation, academic self-efficacy positively predicted learning motivation (*β* = 0.325, *p* < 0.001) and self-control (*β* = 0.285, *p* < 0.001), with effect sizes approaching or reaching medium levels. In the subsequent stage, learning motivation significantly predicted self-control (*β* = 0.301, *p* < 0.001), with a medium effect size, and also exerted a positive effect on online physical education learning engagement (*β* = 0.177, *p* < 0.001), with a small-to-medium effect size. Furthermore, self-control had a significant positive effect on online physical education learning engagement (*β* = 0.314, *p* < 0.001), with a medium effect size (Cohen, 1988). The model accounted for 10.9, 24.1, and 25.4% of the variance in learning motivation, self-control, and online physical education learning engagement, respectively. The total effect of academic self-efficacy on online physical education learning engagement (*β* = 0.326, reported in Table 4) was estimated without mediators; the direct effect after controlling for learning motivation and self-control (*β* = 0.147) is reported in Table 5.

**Table 4:** Structural model path coefficients.

Paths	*β*	SE	C.R.	*p*
Academic self-efficacy → learning motivation	0.325	0.029	10.25	<0.001
Academic self-efficacy → self-control	0.285	0.099	9.222	<0.001
Academic self-efficacy → online physical education learning engagement	0.326	0.088	4.594	<0.001
Learning motivation → self-control	0.301	0.105	10.02	<0.001
Learning motivation → online physical education learning engagement	0.177	0.094	5.545	<0.001
Self-control → online physical education learning engagement	0.314	0.028	9.428	<0.001

*β*, standardized coefficient; SE, standard error; C.R., critical ratio.

**Table 5:** Testing for chain mediation effects.

Paths	Effect	Boot SE	Bootstrap 95%CI	Effect size ratio
Academic self-efficacy → learning motivation → online physical education learning engagement	0.057	0.012	[0.036, 0.081]	17.48%
Academic self-efficacy → self-control → online physical education learning engagement	0.090	0.013	[0.065, 0.116]	27.61%
Academic self-efficacy → learning motivation → self-control → online physical education learning engagement	0.032	0.005	[0.023, 0.044]	9.82%
Total indirect effect between academic self-efficacy and online physical education learning engagement	0.179	0.017	[0.146, 0.214]	54.91%
Direct effect	0.147	0.032	[0.084, 0.210]	45.09%
Total effect	0.326	0.032	[0.263, 0.389]	100%

Effect refers to standardized coefficients; CI, confidence interval; SE, standard error; The total effect (*β* = 0.326, reported in Table 4) was estimated without mediators; the direct effect (*β* = 0.147) is the residual direct effect after controlling for learning motivation and self-control.

In summary, learning motivation and self-control serve as critical mediators between academic self-efficacy and online physical education learning engagement, operating through both independent mediating pathways and a serial mediation mechanism of academic self-efficacy → learning motivation → self-control → online physical education learning engagement.

### Mediation effects

To further examine the mediating roles of learning motivation and self-control in the relationship between academic self-efficacy and online physical education learning engagement, a bias-corrected bootstrap procedure with 5,000 resamples was employed to estimate indirect effects (see Table 5). The results indicated that the sequential mediation pathway—academic self-efficacy → learning motivation → self-control → online physical education learning engagement—was significant (standardized indirect effect = 0.032, 95% CI [0.023, 0.044]). In addition, academic self-efficacy exerted significant indirect effects through each mediator separately. The indirect effect via learning motivation was 0.057 (95% CI [0.036, 0.081]), and the indirect effect via self-control was 0.090 (95% CI [0.065, 0.116]), indicating that both single mediation pathways were statistically significant. After including the mediators, the direct effect of academic self-efficacy on online physical education learning engagement remained significant (standardized direct effect = 0.147, 95% CI [0.084, 0.210]), suggesting partial mediation. Furthermore, the total indirect effect was 0.179, accounting for 54.91% of the total effect, highlighting the substantial role of the mediating mechanisms.

Overall, learning motivation and self-control not only function as independent mediators but also operate sequentially to jointly influence the relationship between academic self-efficacy and online physical education learning engagement.

## Discussion

This study examined the relationship between academic self-efficacy and engagement in online physical education among Chinese university students, and further investigated the mediating roles of learning motivation and self-control. The results indicate that learning motivation and self-control each independently served as mediators in the relationship between academic self-efficacy and engagement in online physical education, and also formed a sequential mediation pathway. These findings are consistent with the proposed hypotheses and contribute to the empirical literature on the psychological processes associated with engagement in online physical education.

### The direct effect of academic self-efficacy on online physical education learning engagement

Consistent with Hypothesis 1 and social cognitive theory (Bandura, 1997), academic self-efficacy was positively associated with engagement in online physical education. As a belief system regarding one’s capability to accomplish learning tasks, academic self-efficacy may play a crucial motivational role in online physical education contexts (Caraway et al., 2003; Frikha et al., 2024). Specifically, learners with higher academic self-efficacy reported greater engagement. First, individuals with high self-efficacy tended to perceive skill challenges as manageable tasks rather than threats, and reported greater willingness to invest cognitive and physical resources. Second, given the absence of immediate feedback in online settings, high self-efficacy was associated with learners’ ability to rely on internal standards to maintain stable engagement. Finally, individuals with high self-efficacy reported greater persistence when facing coordination difficulties (Bandura, 1997).

This study extends the examination of academic self-efficacy to the context of online physical education, addressing the limitation of prior research that has largely focused on general online learning or traditional classroom settings. The embodied nature of physical education creates tension with the virtual characteristics of online environments, and the present findings suggest that academic self-efficacy may help bridge this gap, pointing to the potential importance of fostering learners’ capability beliefs in the design of online physical education.

### The mediating role of learning motivation

The present study found that learning motivation partially mediated the relationship between academic self-efficacy and engagement in online physical education, which is consistent with Hypothesis 2. This finding points to a motivational pathway through which academic self-efficacy is associated with learners’ intrinsic motivational states, which are in turn associated with the investment of cognitive and physical resources.

According to self-determination theory (Ryan and Deci, 2000), academic self-efficacy, as a core component of competence beliefs, may satisfy individuals’ need for competence, which may contribute to the development and maintenance of intrinsic motivation. In the context of online physical education, when learners are confident in their ability to master motor skills, their intrinsic interest and perceived value of participation may be strengthened, which may be associated with higher levels of motivation and sustained engagement. Additionally, the motivational regulation model (Joo et al., 2013) suggests that highly motivated learners are more likely to adopt goal-oriented regulatory strategies when facing physical fatigue or skill-related challenges, focusing on skill improvement rather than immediate discomfort, which may help maintain stable engagement (Pintrich and De Groot, 1990).

Notably, learning motivation only partially mediated this relationship, suggesting that academic self-efficacy may also be directly associated with engagement. This finding aligns with Pintrich’s perspective (2004) that even when perceived task value is low, individuals with high self-efficacy may still persist based on their capability beliefs. In online physical education courses, some students with limited intrinsic motivation may still maintain basic practice through self-regulation if they believe they can meet skill requirements. This “low motivation–high engagement” pattern suggests that instructional design may benefit from addressing both competence beliefs and value construction.

### The mediating role of self-control

The results further indicated that self-control significantly mediated the relationship between academic self-efficacy and engagement in online physical education, which is consistent with Hypothesis 3. This finding points to a regulatory pathway through which academic self-efficacy is associated with engagement, suggesting that learners with higher self-efficacy is associated with their self-control capacity to manage conflicts between immediate temptations and long-term goals, which may help sustain engagement.

This mediating mechanism is particularly salient in the context of online physical education. Duckworth [33] conceptualizes self-control as the capacity to regulate behavior when long-term goals conflict with immediate temptations, and online physical education represents a prototypical context of such goal–temptation conflict. In unsupervised home environments, learners are required to resist distractions while persisting in physically demanding skill practice. Learners with higher academic self-efficacy reported stronger self-control, which may stem from greater certainty regarding effort–outcome expectations. Specifically, when learners believe that practice will lead to skill improvement, the psychological cost of delayed gratification may be reduced, and the depletion of self-control resources may be minimized, which may be associated with greater engagement in behaviors aligned with long-term learning goals (Meng, 2025).

Moreover, the mediating role of self-control is functionally distinct from that of learning motivation. Motivation primarily determines whether engagement is initiated, whereas self-control governs whether engagement can be sustained. By confirming the significance of both pathways, this study provides empirical evidence consistent with a dual-process model of motivation and control in online physical education.

### The sequential mediating roles of learning motivation and self-control

The present study found that learning motivation and self-control jointly constituted a significant sequential mediation pathway between academic self-efficacy and engagement in online physical education, which is consistent with Hypothesis 4. This pathway—from academic self-efficacy to learning motivation to self-control and ultimately to engagement—reflects a progressive mechanism through which capability beliefs are associated with behavioral investment. Specifically, academic self-efficacy is associated with learners’ motivational states, which are in turn associated with self-control resources that may help sustain engagement over time.

From a theoretical perspective, this sequential model aligns with and extends prior research in several ways. The positive association between academic self-efficacy and learning motivation is consistent with previous findings (Meng, 2025; Cai and Jia, 2020). More importantly, the present study indicates that learning motivation is associated with engagement through the regulatory function of self-control. This motivation–control–engagement pathway is consistent with self-regulated learning theory, which posits that motivation initiates action, whereas volition and control sustain it (Gollwitzer, 1990). Attribution theory also suggests that individuals’ interpretations of success and failure—particularly in terms of ability beliefs and perceived controllability—are associated with their patterns of behavioral engagement (Weiner, 1985). The sequential pathway identified in this study may be viewed as a concrete manifestation of this mechanism. Finally, this study provides empirical evidence consistent with Martin’s Motivation and Engagement Wheel model (Martin, 2007), with self-control appearing to serve as a critical bridging mechanism between adaptive cognition and adaptive behavior.

Notably, although our findings are associated with the hypothesized directional relationships, alternative theoretical interpretations should also be acknowledged. It is also plausible that engaged students may develop higher self-efficacy through successful mastery experiences (Bandura, 1997). Regarding the role of self-control, alternative perspectives suggest that self-control may precede and be associated with motivation by enabling individuals to resist immediate temptations and maintain focus on long-term goals, which may be associated with sustained motivation over time (Duckworth, 2011). Reciprocal relationships among these variables are also conceivable, as higher engagement may be associated with motivation and self-control through positive feedback. These alternative pathways suggest that caution is warranted exercised when interpreting the present findings.

### Interpretation of model explanatory power

The model accounted for 10.9, 24.1, and 25.4% of the variance in learning motivation, self-control, and online physical education learning engagement, respectively. These *R*^2^ values range from moderate to large in magnitude according to Cohen’s guidelines [60], indicating adequate explanatory power for detecting the hypothesized mediating effects. Given the multifaceted nature of learning engagement, which is shaped by individual, contextual, and instructional factors, it is not surprising that a single set of psychological predictors explains a moderate proportion of its variance. This suggests that other unmeasured variables, such as course design features, perceived social support, or prior physical activity experience, may also contribute to engagement.

### Theoretical contributions and practical implications

The theoretical contributions of this study can be summarized in three aspects. First, this study extends the application of academic self-efficacy to the context of online physical education—unlike general online learning, online physical education requires learners to sustain physical effort and skill practice in the absence of on-site guidance, and capability beliefs appear particularly critical in bridging the gap between virtual instruction and physical execution. Second, it identifies the distinct roles of learning motivation (initiation) and self-control (maintenance) in engagement, and provides evidence consistent with a dual-process model of motivation and control. Third, it identifies and validates a sequential mediation model (academic self-efficacy → learning motivation → self-control → engagement), offering an integrative theoretical framework for understanding how motivational activation and regulatory maintenance may jointly shape sustained behavioral investment.

From a practical perspective, educators could prioritize the cultivation of learners’ capability beliefs by implementing staged skill goals, multi-angle demonstrations, and data-driven feedback. For students with low motivation but high self-efficacy, instructional design could emphasize the relevance of physical education to health and wellbeing which may be associated with intrinsic motivation. Given the potential role of self-control in sustaining engagement, online platforms could integrate self-monitoring tools that may help students manage distractions in home environments and maintain consistent learning behaviors.

### Limitations and future directions

Although this study identified multiple mediating pathways between academic self-efficacy and engagement in online physical education, several limitations should be acknowledged and addressed in future research. First, the cross-sectional design limits the ability to infer causal relationships. Although the proposed sequential mediation model was theoretically grounded, bidirectional or reciprocal relationships among the variables cannot be ruled out. Future studies could employ longitudinal designs or experience sampling methods to capture dynamic changes over time and more rigorously test causal directions. In addition, this study did not collect information on participants’ socioeconomic status, prior online learning experience, physical activity level, or frequency of online physical education participation. These factors may be associated with access to digital resources, home learning environments, and physical activity opportunities, and may be associated with learning engagement. Second, all data were derived from self-report measures, which may introduce common method bias and may influence the accuracy of parameter estimates. Although statistical tests were conducted to assess common method bias, future research could incorporate multi-source data, such as behavioral logs from learning platforms, teacher evaluations, or peer assessments, to enhance measurement validity. Third, this study focused on learning motivation and self-control as mediators; however, academic self-efficacy may also be associated with engagement through other mechanisms, such as learning strategies or perceived social support. Future research could expand the mediating network to develop a more comprehensive model of engagement in online physical education. Fourth, this study did not collect class-level information and did not account for the nested structure of students within classes or schools, which may result in biased standard error estimates. Future research could employ multilevel designs to examine whether these relationships vary across classes or schools. Finally, the sample was drawn from only two universities in China, and detailed information on the specific types of online physical education courses in which students were enrolled was not collected. This may limit the generalizability of the findings to other countries, age groups, or course formats. Future research could extend the sample to diverse cultural and educational contexts and incorporate course type as a contextual variable to further examine the cross-contextual applicability of the proposed model.

## Conclusion

Grounded in social cognitive theory, expectancy–value theory, and the strength model of self-control, this study examined whether academic self-efficacy was associated with engagement in online physical education among college students, and whether learning motivation and self-control mediated this association. The results indicate that academic self-efficacy was directly associated with engagement, and was also indirectly associated with engagement via learning motivation and self-control, including a sequential pathway from motivation to self-control. From the dual perspectives of behavioral initiation and maintenance, these findings are consistent with the theoretically proposed sequential mediation model and contribute to understanding the psychological processes associated with engagement in online physical education contexts.

## Data Availability

The raw data supporting the conclusions of this article will be made available by the authors, without undue reservation.
